# CRISPR library screening to develop HEK293-derived cell lines with improved lentiviral vector titers

**DOI:** 10.3389/fgeed.2023.1218328

**Published:** 2023-07-13

**Authors:** Brian J. Iaffaldano, Michael P. Marino, Jakob Reiser

**Affiliations:** Division of Cellular and Gene Therapies, Center for Biologics Evaluation and Research, U.S. Food and Drug Administration, Silver Spring, MD, United States

**Keywords:** lentiviral vectors, CRISPR library screening, vector titers, gene knockout, gene activation

## Abstract

Lentiviral (LV) vectors have emerged as powerful tools for treating genetic and acquired human diseases. As clinical studies and commercial demands have progressed, there has been a growing need for large amounts of purified LV vectors. To help meet this demand, we developed CRISPR library screening methods to identify genetic perturbations in human embryonic kidney 293 (HEK293) cells and their derivatives that may increase LV vector titers. Briefly, LV vector-based Human CRISPR Activation and Knockout libraries (Calabrese and Brunello) were used to modify HEK293 and HEK293T cells. These cell populations were then expanded, and integrated LV vector genomes were rescued by transfection. LV vectors were harvested, and the process of sequential transduction and rescue-transfection was iterated. Through this workflow, guide RNAs (gRNAs) that target genes that may suppress or enhance LV vector production were enriched and identified with Next-Generation Sequencing (NGS). Though more work is needed to test genes identified in this screen, we expect that perturbations of genes we identified here, such as *TTLL12*, which is an inhibitor of antiviral innate immunity may be introduced and multiplexed to yield cell lines with improved LV vector productivity.

## 1 Introduction

Lentiviral (LV) vectors have been utilized in numerous clinical trials to deliver transgenes into primary cells *ex vivo*, including T cells and hematopoietic stem cells (Milone and O’Doherty, 2018). Recently approved CAR T cell gene therapy products consisting of autologous patient T cells transduced with LV vectors include, Kymriah (tisagenlecleucel) to treat B-cell acute lymphoblastic leukemia (B-ALL) (Liu et al., 2017), Breyanzi (lisocabtagene maraleucel) to treat large B-cell lymphoma (LBCL) (Abramson et al., 2020), as well as Abecma (idecabtagene vicleucel) and Carvykti (ciltacabtagene autoleuce) to treat multiple myeloma (Munshi et al., 2021; U.S. Food and Drug Administration, 2022a). Zynteglo (betibeglogene autotemcel) consists of genetically modified autologous CD34^+^ cells containing haematopoietic stem cells (HSC) transduced with LV vectors bearing the β^A−T87Q^-globin transgene for the treatment of patients with transfusion-dependent beta-thalassemia (Schuessler-Lenz et al., 2020; U.S. Food and Drug Administration, 2022b). Skysona (elivaldogene autotemcel) is a LV vector-based gene therapy involving of genetically modified autologous CD34^+^ cells to treat boys with early, active cerebral adrenoleukodystrophy (CALD) (U.S. Food and Drug Administration, 2022c).

LV vector titers are often insufficient, and production is costly to scale (Perry and Rayat, 2021). The cells used to produce LV vectors are typically based on human embryonic kidney (HEK) 293 cells and its derivative cell lines such as HEK293T. Such cells have been rationally modified to further improve titers and suit specific vectors (Sosale et al., 2016, 47; Milani et al., 2017; Zhang et al., 2017; Hu et al., 2018; Ozog et al., 2018; Shirley et al., 2020). Notably, a multi-gene knockout clone of 293T cells resulted in a 7-fold increase in LV titers of complex vectors (Han et al., 2021). While thus far, rational approaches have been utilized to improve LV production, recently, an unbiased genome-wide CRISPR activation screen has been used to increase viral titer and infectious units of different adeno-associated virus (AAV) vector serotypes over 3-fold (Barnes et al., 2021); however, results of unbiased CRISPR library screens to increase LV vector titers have not been reported to date. However, OhAinle *et al.*, have used a CRISPR library approach, where 15,348 gRNAs implicated in interferon response were screened by rescuing vectors containing Cas9 and gRNA with wild type HIV (OhAinle et al., 2018). Along similar lines, here, we have detailed a transfection-based workflow that can be used to identify genetic perturbations that can improve the ability of cells to produce lentiviral vectors. Using this approach, we identified candidate genes to be knocked out or overexpressed.

Using this modified knockout library, we first elected to conduct a pilot library screening experiment in HEK293 cells as a proof of concept, using a genome wide, LV vector-based CRISPR knockout library referred to as the Brunello library (Doench et al., 2016; Sanson et al., 2018). To do this, we developed an approach relevant to the production of LV vectors by introducing the entire Brunello gRNA library into HEK293 cells and rescuing integrated LV vector genomes encoding specific gRNAs by transient transfection. Using the smaller scale of the pilot screening we were able to generate sequence data for each cycle of rescue transfection and transduction, to observe changes in gRNA abundance over time. We then went on to broaden our methodology, by conducting full scale screenings using both the Brunello knockout library and Calabrese activation library in HEK293T cells (Lin et al., 2014). Through this approach, we observed the enrichment over time of gRNAs targeting defined genes, which introduce genetic perturbations that may increase LV titers.

## 2 Materials and methods

### 2.1 Construction of vectors and CRISPR libraries

LentiCRISPR v2 (Addgene Plasmid #52961, kindly provided by Feng Zhang) (Sanjana et al., 2014) was used as the backbone for a non-self-inactivating CRISPR library. This plasmid was digested with *BamH*I and *Pme*I and the large plasmid backbone fragment was gel extracted. A wild type LTR was added *via* a gBlock WPRE wt3′LTR sequence (Supplementary Figure S1) and a marker sequence, encoding either 2A Puro or 2 GFP, was added back via NEBuilder^®^ HiFi DNA Assembly (New England Biolabs, Ipswich, MA). The 2A Puro fragment was amplified from LentiCRISPR v2 using the primers Hifi puro F (5′ gat​tac​aaa​gac​gat​gac​gat​aag​gGA​TCC​GGC​GCA​ACA​AAC​TTC 3′) and Hifi puro R (5′ cca​gag​gtt​gat​tGT​CGA​CTT​AAC​GCG​TTC​AG 3′). The 2a gfp fragment was amplified from TLCV2 (Addgene Plasmid #87360, kindly provided by Adam Karpf) (Barger et al., 2019) using the primers Hifi EGFP F (5′gat​tac​aaa​gac​gat​gac​gat​aag​gGA​TCC​GGA​GAG​GGC​AGA​G 3′) and Hifi EGFP R (5′cag​agg​ttg​att​gtc​gac​tta​acg​cGT​TTA​CTT​GTA​CAG​CTC​GTC​C 3′). PCR products were column purified prior to assembly. Assembly reactions were transformed into NEB^®^ Stable Competent *E. coli* (New England Biolabs, Ipswich, MA) produced using a Zymo Mix & Go! *E. coli* Transformation Kit (Zymo Research, Irvine, CA). Colonies were verified by digestion with *BamH*I and *Pme*I, as well as Sanger sequencing. The assembled vector conferring puromycin resistance was designated as LentiCRISPR wt LTR Puro. Vectors were also constructed for a two-part CRISPR library system capable of gene knockout, activation and inhibition. This was achieved by modifying lentiGuide-Puro (Addgene Plasmid #52963, kindly provided by Feng Zhang) (Sanjana et al., 2014), pXPR_502 (Addgene Plasmid #96923, kindly provided by John Doench and David Root) (Sanson et al., 2018) and pXPR_050 (Addgene Plasmid #96925, kindly provided by John Doench and David Root) (Sanson et al., 2018), respectively. In this case, the wild type LTR sequence was introduced by PCR amplifying a fragment from pHR-CMV-nlsCRE (Addgene Plasmid #12265, kindly provided by Didier Trono) (Cudré-Mauroux et al., 2003), using the primers 3′LTR F (5′ taa​gac​caa​tga​ctt​aca​agG​CAG​CTG​TAG​ATC​TTA​GC 3′) and 3′LTR R (5′ atg​aca​tga​act​act​ata​cgT​ACT​GCT​AGA​GAT​TTT​CCA​C 3′). Backbones were amplified from lentiGuide-Puro, pXPR_502 and pXPR_050 using the primers Library backbone F (5′ CGT​ATA​GTA​GTT​CAT​GTC​ATC 3′) and Library backbone R (5′ CTT​GTA​AGT​CAT​TGG​TCT​TAA​AG 3′). Products were amplified using Q5^®^ High-Fidelity DNA Polymerase (New England Biolabs, Ipswich, MA). Fragments were gel purified and assembled using NEBuilder^®^ HiFi DNA Assembly (New England Biolabs, Ipswich, MA).

To alter the plasmid backbone of the Brunello library, the vector LentiCRISPR wt LTR Puro was digested with *BsmB*I and the large fragment was gel extracted. The Brunello library was acquired from Addgene as pooled plasmids (Addgene Pooled Library #73179, kindly provided by David Root and John Doench) (Doench et al., 2016; Sanson et al., 2018). The region containing the gRNA sequences was amplified using the primers Oligo-Fwd zhang 2017 (5′ GTA​ACT​TGA​AAG​TAT​TTC​GAT​TTC​TTG​GCT​TTA​TAT​ATC​TTG​TGG​AAA​GGA​CGA​AAC​ACC 3′) and Oligo-Knockout-Rev zhang 2017 (5′ ACT​TTT​TCA​AGT​TGA​TAA​CGG​ACT​AGC​CTT​ATT​TTA​ACT​TGC​TAT​TTC​TAG​CTC​TAA​AAC 3′) (Joung et al., 2017). Eight 25 µL PCR reactions were carried out for 18 amplification cycles, using a total 50 ng of input plasmid DNA. The PCR products were pooled and gel purified. The modified backbone and gRNA fragments were assembled with an excess for gRNA inserts using NEBuilder^®^ HiFi DNA Assembly (New England Biolabs, Ipswich, MA). The assemblies were electroporated into Endura™ Competent Cells (Lucigen Corporation, Middleton, WI), using 1.0 mm cuvettes with a Gene Pulser Xcell (Bio-Rad Laboratories, Hercules, CA) and parameters of 10 µf, 600 Ω and 1,800 V. Each electroporation consisted for 30 µL of competent cells and 40 ng of library plasmid DNA. Cells were allowed to recover in 2.5 mL of the provided recovery media in an incubator-shaker set to 37°C for 1 hour. After recovery, the electroporated cells were pooled and serial dilutions were plated. The remainder of cells were grown in 500 mL of liquid culture overnight at 30°C. Colony counts of the plated serial dilutions indicated that the liquid culture consisted of approximately three million colony forming units. Plasmid DNA was extracted from the 500 mL culture using a ZymoPURE™ II Plasmid Maxiprep Kit (Catalog #D4203, Zymo Research, Irvine, CA). To clone the Brunello library in a wild-type LTR lentiGuide-Puro (Addgene Plasmid #52963, kindly provide by Feng Zhang) (Sanjana et al., 2014) backbone, PCRs, digestions and HiFi reactions were conducted as described above. The gRNA from the Calabrese library were cloned into a wild type LTR backbone similarly; however, the reverse primer Calabrese Reverse (5′ GAG​AGG​AGC​GAC​GCC​ATA​TCG​TCT​GCT​CCC​TCG​TAT​TCG​CAG​CAT​AGC​TCT​TAA​AC 3’) was used for PCR. The transformations for full scale two-part vector screening of Brunello and Calabrese part A and Calabrese part B were scaled as described above, with approximately 24 electroporations for each library and plated on 12,500 cm^2^ bioassay dishes for each library. Colonies were harvested by scraping with LB broth, pelleting and extracting via ZymoPURE™ II Plasmid Maxiprep Kit (Catalog #D4203, Zymo Research, Irvine, CA), as described in Joung et al., 2017 (Joung et al., 2017). At least 3 × 10^9^ colony forming units were collected for each library. The new plasmids are available through Addgene; they include: lenticrispr-wt-ltr-puro (Addgene Plasmid #173428), lentiguide-puro-wt-ltr (Addgene Plasmid #182635), pxpr_050-wt-ltr (Addgene Plasmid #182646), and pxpr_502-wt-ltr (Addgene Plasmid #182647).

To construct doxycycline-inducible gene knockout vectors, the TLCV2 plasmid was digested with *BsmB*I. The linearized plasmid was gel extracted and ligated to gene specific oligo-duplexes formed using the primers listed in Supplementary Table S1. Plasmids were transformed as previously described and colonies were miniprepped and Sanger sequenced using the primer hU6-F (5′ GAG​GGC​CTA​TTT​CCC​ATG​ATT 3’). All restriction enzymes used in this study were from New England Biolabs. All CRISPR nucleases used were S. pyogenes Cas9 (SpCas9).

### 2.2 Cell lines

Human embryonic kidney (HEK) 293T cells (CRL-11268; American Type Culture Collection [ATCC], Manassas, VA) and HEK293 cells (Catalog Number 103, NIH AIDS Reagent Program, Germantown, MD) were grown in Dulbecco’s Modified Eagle’s Medium (DMEM) containing high glucose (4.5 g/L), 2 mM L-glutamine, 10% heat inactivated fetal bovine serum (FBS), 100 U/mL penicillin and 100 μg/mL streptomycin. Cell culture reagents were purchased from Gibco (Thermo Fisher Scientific, Waltham, MA).

### 2.3 Preparation of CRISPR libraries using a lentiviral vector bearing an intact 3’ LTR

The cloned wtLTR Brunello and Calabrese plasmid libraries were first transfected into HEK293T cells. HEK293T cells were plated in four 225 cm^2^ flasks at 1 × 107 cells per flask 1 day prior to transfection. These flasks were transfected with library vector, third generation helper plasmids pCD/NL-BHΔ1 (Addgene Plasmid #41791) (Ou et al., 2012) and pCMV-Rev (NIH HIV Reagent Program ARP-1443) and VSV-G envelope plasmid pCEF-VSV-G (Addgene Plasmid #41792) (Ou et al., 2012), using polyethylenimine (PEI) transfection as described (Kuroda et al., 2009). Briefly, 12 µg of library vector, 9.6 µg of pCD/NL-BHΔ1, 12.48 µg of pCMV-Rev, and 4.08 µg of envelope was diluted with 2.4 mL of 150 mM NaCl and mixed with an equal volume of PEI (Catalog # 24765, Polysciences, Warrington, PA) per flask. This DNA: PEI mixture was incubated for 10min at RT and added to each flask. LV vector stocks were collected and concentrated as described (Kutner et al., 2009), and were titrated by puromycin selection ***.*** In order to titrate, 70,000 HEK 293 cells were seeded into 6 well plates 1 day prior to transduction. Cells were transduced with varying doses of LV vector samples as described above. The medium was changed the following day. After one additional day, cells were trypsinized and evenly split into media with or without 1 μg/mL puromycin. In parallel, non-transduced cells were added to selection media. After 3 days non-transduced cells were completely killed. Transduced cells were counted with or without puromycin selection to estimate LV vector titers. Approximately 100,000 cells were initially treated with LV vectors. After selection cells were trypsinized and counted by Cellometer^®^ Auto T4 Bright Field Cell Counter or Celigo Imaging Cytometer (Nexcelom Bioscience LLC, Lawrence, MA) and LV titers were calculated. For two vector screening using the Brunello and Calabrese libraries, HEK293T cells were used for titration instead of HEK293 cells.

### 2.4 Carrying out rounds of pilot Brunello CRISPR library screening

To conduct the pilot Brunello screening, the concentrated LV library was then used to transduce HEK293 cells as described above. Approximately 50 million cells were transduced in four 150 cm^2^ flasks with a multiplicity of infection of approximately 0.4, based on the small-scale functional titration described above. After overnight transduction, the medium was replaced. The next day, cells were trypsinized and the cells from all four flasks and seeded into four new flasks containing 0.4 μg/mL puromycin (Thermo Fisher catalog # A1113803). After 2 days, the cells were trypsinized and pelleted. Half of these cells were frozen and reserved for genomic DNA extraction while the remaining cells were divided between four 225 cm^2^ flasks. After the cells reached approximately 80% confluency, they were PEI transfected with 32.64 µg of pCEF-VSV-G and 65.28 µg of second generation pCD/NL-BH*ΔΔΔ packaging plasmid (Addgene plasmid #17531) (Zhang et al., 2004). 32.64 µg of pCEF-VSV-G and 65.28 µg of pCD/NL-BH*ΔΔΔ was diluted with 9.6 mL of 150 mM NaCl and mixed with an equal volume of PEI. This DNA: PEI mixture was incubated for 10 min at RT and divided equally between each flask. The medium was replaced the morning after transfection, and LV vectors were harvested after an additional 2 days. Note that the amount of pCEF-VSV-G and pCD/NL-BH*ΔΔΔ are compared to a standard protocol, which maintains the DNA:PEI ratio as a vector plasmid is omitted. As before, LV vectors were concentrated by ultracentrifugation and titrated (Kutner et al., 2009). After titration these preparations could then be used to transduce fresh HEK293 cells that have not been exposed to library sequences. These cells could then in turn be selected and transfected. This process was repeated for 8 rescue cycles to allow differences in gRNA abundances to be compounded over time.

### 2.5 Carrying out rounds of large-scale Brunello and Calabrese CRISPR library screening

HEK293T cells carrying Cas9 vectors were first made by transducing with lentiCas9-Blast (Addgene Plasmid #52962, kindly provided by Feng Zhang) (Sanjana et al., 2014) or lenti dCAS-VP64_Blast (Addgene Plasmid #61425, kindly provided by Feng Zhang) (Konermann et al., 2015), for screening with the Brunello and Calabrese libraries, respectively. HEK293T cells were transduced with a multiplicity of infection of 0.4, based on the small-scale functional titration described above. Cells were then selected with 4 μg/mL blasticidin (Thermo Fisher catalog # A1113903). Cells were then seeded in 150 cm^2^ flasks, with each flask receiving approximately 12 million cells and selected with puromycin as described above. Each library, Brunello, Calabrese A, and Calabrese B was screened in triplicate, with each Brunello experimental unit consisting of four flasks and each Calabrese experimental unit consisting of three flasks, for a total of 30 concurrent 150 cm^2^ flasks. Iterative rounds of rescue transfection followed by transduction as described above were then conducted, with minimal gRNA population sizes of 40 million for Brunello experimental units and 30 million for Calabrese experimental units maintained. Notably, as HEK293T cells yield higher LV titers than HEK293 cells, concentration by ultracentrifugation was not required for this workflow. However, in order to prepare viral RNA following the eighth round of rescue, ultracentrifugation was used and RNA from at least 50 million transducing units was carried forward to PCR.

### 2.6 Characterization of pilot CRISPR libraries

The primers Brunello Seq F (5′ TTG​TGG​AAA​GGA​CGA​AAC​ACC​G 3′) and Brunello Seq R (5′ CCA​ATT​CCC​ACT​CCT​TTC​AAG​ACC​T 3’) were used to amplify the region containing the gRNA sequences. For plasmid templates, Q5^®^ High-Fidelity DNA Polymerase (Catalog #M0491, New England Biolabs, Ipswich, MA) was used for 20 cycles of amplification in four 50 µL reactions. To sequence vector RNAs, RNA was extracted from concentrated LV vector stocks, each representing approximately 40 million transducing units (TUs), using a Quick-RNA Viral Kit (Catalog #D4033, Zymo Research, Irvine, CA). Half of the extracted RNA was used in four 50 µL OneTaq^®^ One-Step RT-PCR reactions (Catalog #E5315, New England Biolabs, Ipswich, MA) with 20 cycles of amplification. In all cases, products were visualized on a 2% agarose gel and then column purified (Catalog #D4033, Zymo Research, Irvine, CA). Purified products were sent for CRISPR sequencing at the Massachusetts General Hospital DNA Core. FastQ files were then analyzed to determine the abundance of library members using a modified python script, with KEY_REGION_START = 10 and KEY_REGION_END = 35 (Joung et al., 2017).

### 2.7 Characterization of large-scale CRISPR libraries

Sequencing libraries were generated as described by (Joung et al., 2017). The ultramers identified as NGS-Fwd #1-10 and NGS-KO-Rev #one to eight were used for both the Brunello and Calabrese libraries. When viral RNA was used as a template, OneTaq^®^ One-Step RT-PCR reactions (Catalog #E5315, New England Biolabs, Ipswich, MA) was used for 12 cycles, with each experimental unit amplified in four separate 50 µL reactions. PCR products were then concentrated and gel extracted as described and sequenced using the NovaSeq platform (Illumina, San Diego, CA) (Joung et al., 2017). Approximately 200 million reads were generated for each experimental unit, as well as wild type LTR Brunello and Calabrese libraries. The reads were counted and gRNA, gene-level analysis and Gene Set Enrichment Analysis (GSEA) (Mootha et al., 2003; Subramanian et al., 2005) was conducted with default parameters using the MAGeCK(Li et al., 2014) toolkit hosted on usegalaxy. eu (The Galaxy Community, 2022).

### 2.8 Development of bulk knockout cell populations using doxycycline inducible lentiviral vectors

Vectors harboring gRNA targeting control safe harbor sites or specific exons identified in the library screening were transduced into HEK293 cells in a 6-well format at a MOI of less than 0.1 using transduction methods described above. Cells were passaged using media containing 0.4 μg/mL puromycin. After 3 days, cells were transferred into media containing puromycin and 1 μg/mL doxycycline. They were maintained in this medium for 1 week. The presence of gene editing was confirmed for the *TRIP12* locus, as described below. Cells were then grown in 75 cm^2^ flasks with medium containing 0.2 μg/mL puromycin for an additional week.

### 2.9 Quantification of editing frequency

The targeted *TRIP12* locus was PCR amplified using the primers TRIP12 Long F (5′ TGA​GGT​TGG​GAG​TTG​GAG​AC 3′) and TRIP12 Long R (5′ ACC​GAT​CCT​GAA​CTG​GAG​TG 3′). The amplicons were cleaned using an Exo-CIP™ Rapid PCR Cleanup Kit (New England Biolabs, Ipswich, MA) and sequenced using an internal primer, TRIP12 Seq (5′ AGC​TAG​TAA​TAC​TGC​TGG​AGA​GGT 3′). The frequency of indel formation was evaluated by using ICE Analysis (https://ice.synthego.com/#/). Alternatively, editing frequency was quantified by generating a 198 bp PCR product using the primers, TRIP12 NGS F (5′ TTG​ATG​AAA​GTT​GAA​ATT​GAG​CA 3′) and TRIP12 NGS R (5′ CAC​TTT​TCC​ATT​TTC​TGT​GGA3’). Such products were column purified and sent for CRISPR sequencing at the Massachusetts General Hospital DNA Core. Editing frequency was then quantified by using CRISPResso2 (Clement et al., 2019). An example of an agarose gel-based assay to measure indel formation in clonal populations is also provided in Supplementary Figure S2.

### 2.10 Quantification of cell line LV titers

Prior to characterization of LV vector productivity, HEK 293 cells were cultured in 75 cm^2^ flasks. These cells were then plated in a 6-well or 24-well format and tested for LV productivity using an EGFP vector, as described above for third generation transfection (Kuroda et al., 2009). EGFP positive cells were counted by flow cytometry or Celigo Imaging Cytometer (Nexcelom Bioscience LLC, Lawrence, MA).

### 2.11 Statistical analysis

Pairwise comparison was conducted using a two-tailed Student’s t-test. Significant differences (*p* < 0.05) between two treatments are indicated by asterisks.

## 3 Results

### 3.1 Establishment of CRISPR library screening workflow

CRISPR libraries are commonly distributed in a LV vector context; however, they are typically established using third generation, self-inactivating LV vector backbones with the U3 regions of the vector long terminal repeats (LTRs) deleted. As the workflow we implemented requires iterative rounds of vector production and transduction, we first modified an existing LV vector-based CRISPR knockout plasmid library (Doench et al., 2016; Sanson et al., 2018), by sub-cloning it into a modified LV vector backbone that harbors an intact 3’ LTR to facilitate rescue of infectious LV vector particles.

**FIGURE 1:**
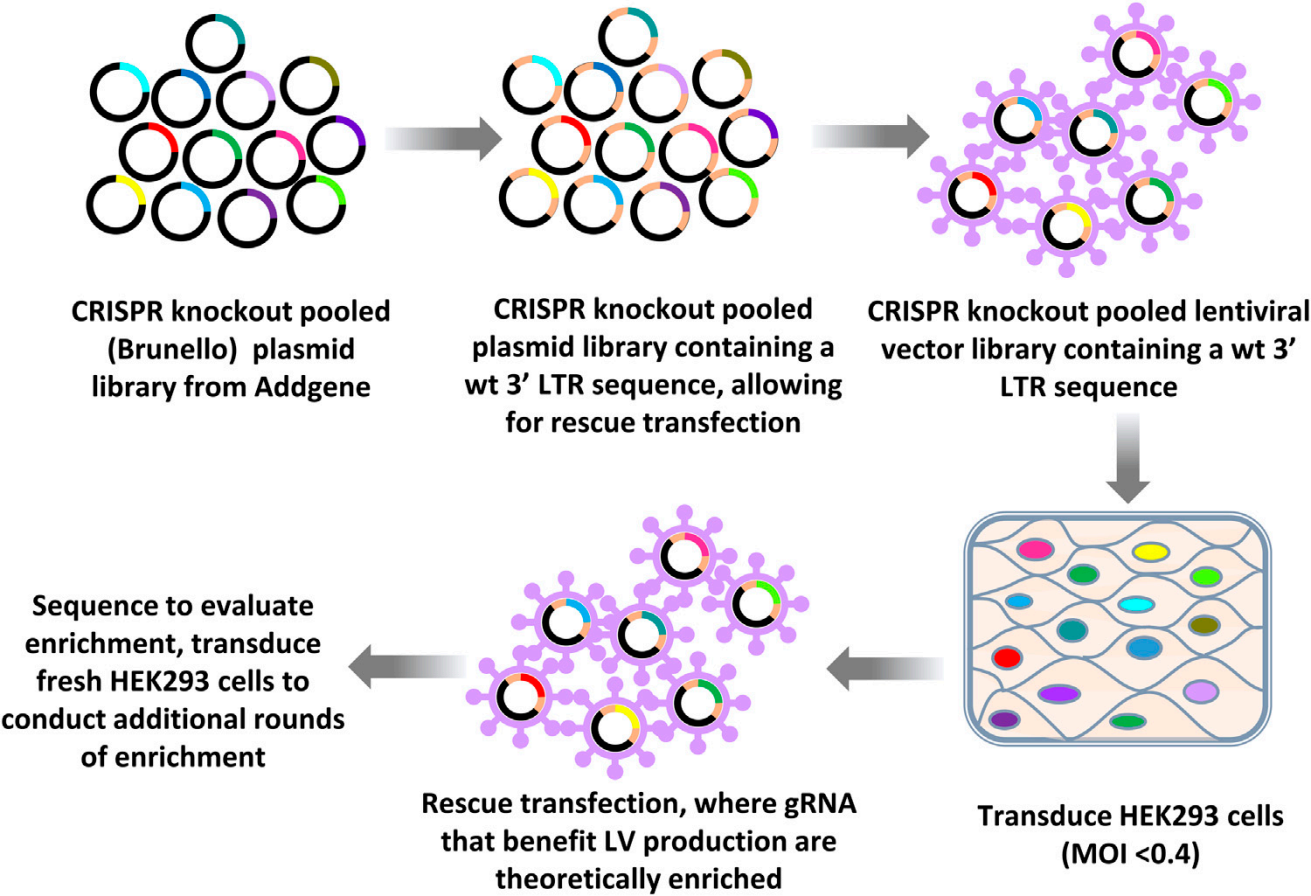
Workflow of CRISPR knockout library screening to identify genomic targets affecting LV vector production. The CRISPR knockout (Brunello) plasmid library (top panel left) was first subcloned into a LV vector plasmid backbone containing an intact (wild type) 3′ LTR (top panel middle). LV vectors were produced by transfecting this vector plasmid library plus plasmids encoding LV packaging functions and VSV-G into HEK293T cells (top right panel) to generate LV vector particles. The resulting LV vector library was then used to transduce HEK293 cells at an MOI of less than 0.4. LV vector particles were rescued from transduced cells by rescue transfection using LV packaging and VSV-G-encoding plasmids. This process of transduction followed by rescue transfection was iterated. The gRNAs encoded by the LV vectors were sequenced by performing RT-PCR using vector RNA from concentrated vector stocks or by using plasmid DNA as a template. PCR products were then sequenced.

An outline of the LV vector production CRISPR knockout LV vector library screening workflow approach using knockout libraries is provided in Figure 1. After one initial round of LV vector production, iterative rounds of LV production were carried out by rescue transfection using LV packaging and VSV-G-encoding plasmids to rescue integrated vector genomes. Fresh HEK293 cells, which were not yet exposed to the CRISPR library, were then transduced with a multiplicity of infection (MOI) of less than 0.4, such that on average, transduced cells only contained a single gRNA, targeting a single gene. During this pilot screening experiment using HEK293 cells, titers for the rescued LV vectors typically ranged from 5 to 8 × 10^5^ transducing units (TU)/ml and the total yields of rescued vectors were typically greater than 10^8^ TU.

### 3.2 Characterization of a modified knockout library

**FIGURE 2:**
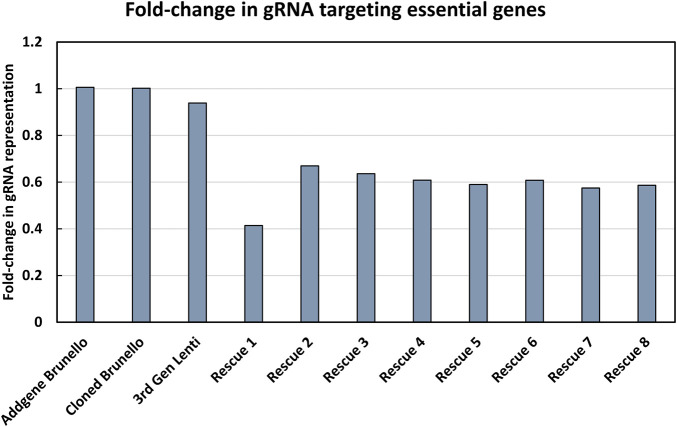
Characterization of the modified LV vector CRISPR knockout library. The fold-change in gRNAs targeting essential genes was determined. The *y*-axis shows the fold-change in gRNA representation of essential genes, where a value of 1 indicates that the number of reads in the library mapping to essential genes is proportional to their abundance in the library. The *x*-axis shows the progress of the workflow, where each name represents a pool of PCR products that was sequenced, starting from the initial knockout plasmid library from Addgene (referred to as Addgene Brunello), the plasmid library involving a LV vector plasmid with a wild type LTR (referred to as “Cloned Brunello”), lentiviral particles produced by using the LV vector plasmid library (referred to as “third Gen Lenti”) and ending with iterative rounds of viral rescue (referred to as “Rescue 1-8”).

**FIGURE 3:**
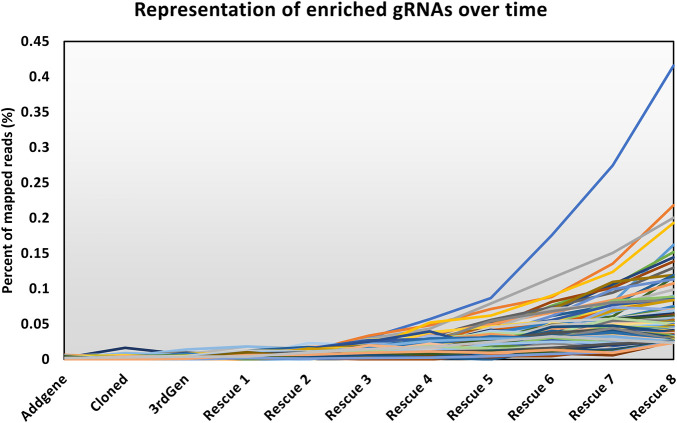
Abundance of enriched gRNAs over time. The line chart shows the representation of the top 255 gRNAs throughout the screen, where the *y*-axis shows the percentage of the mapped reads attributed to an individual gRNA among all the mapped reads. The *x*-axis shows the progress of the workflow as described in Figure 1.

The modified LV vector library was characterized by calculating the abundance of gRNAs targeting essential genes for a pool of 1,734 genes considered essential for cell survival (Figure 2) (Blomen et al., 2015). Our expectation was that gRNAs targeting genes considered essential for cell survival would decrease in abundance if the library was functional. In a plasmid context of the library, the proportion of gRNAs targeting essential genes did not change, and thus met expectations, as CRISPR reagents were not yet expressed or acting on mammalian cells. The number of gRNA reads mapping to essential genes was proportional to their representation of the total gRNA population. The gRNAs targeting essential genes were modestly reduced when vectors were produced using the LV vector-based knockout library containing wild type (wt) LTR sequences and transient transfection of third-generation LV packaging constructs in HEK293T cells. In LV vector populations generated by rescue transfection using LV packaging and VSV-G-encoding plasmids, gRNAs targeting essential genes were reduced by roughly 50%. The regular enrichment of select gRNAs was observed over 8 rounds of LV vector rescue (Figure 3). Normalized read counts for pilot Brunello screening are available in Supplementary Table S2. Having observed that our modified library was active in HEK293 cells, as the abundance of gRNA targeting essential genes was reduced and that select gRNAs regularly increased in abundance over time, we went on to pursue additional validation of identified gRNAs, as well as conduct additional library screening experiments on a larger scale.

### 3.3 Characterization of library hits in HEK293 cells for impact on lentiviral vector titers

**FIGURE 4:**
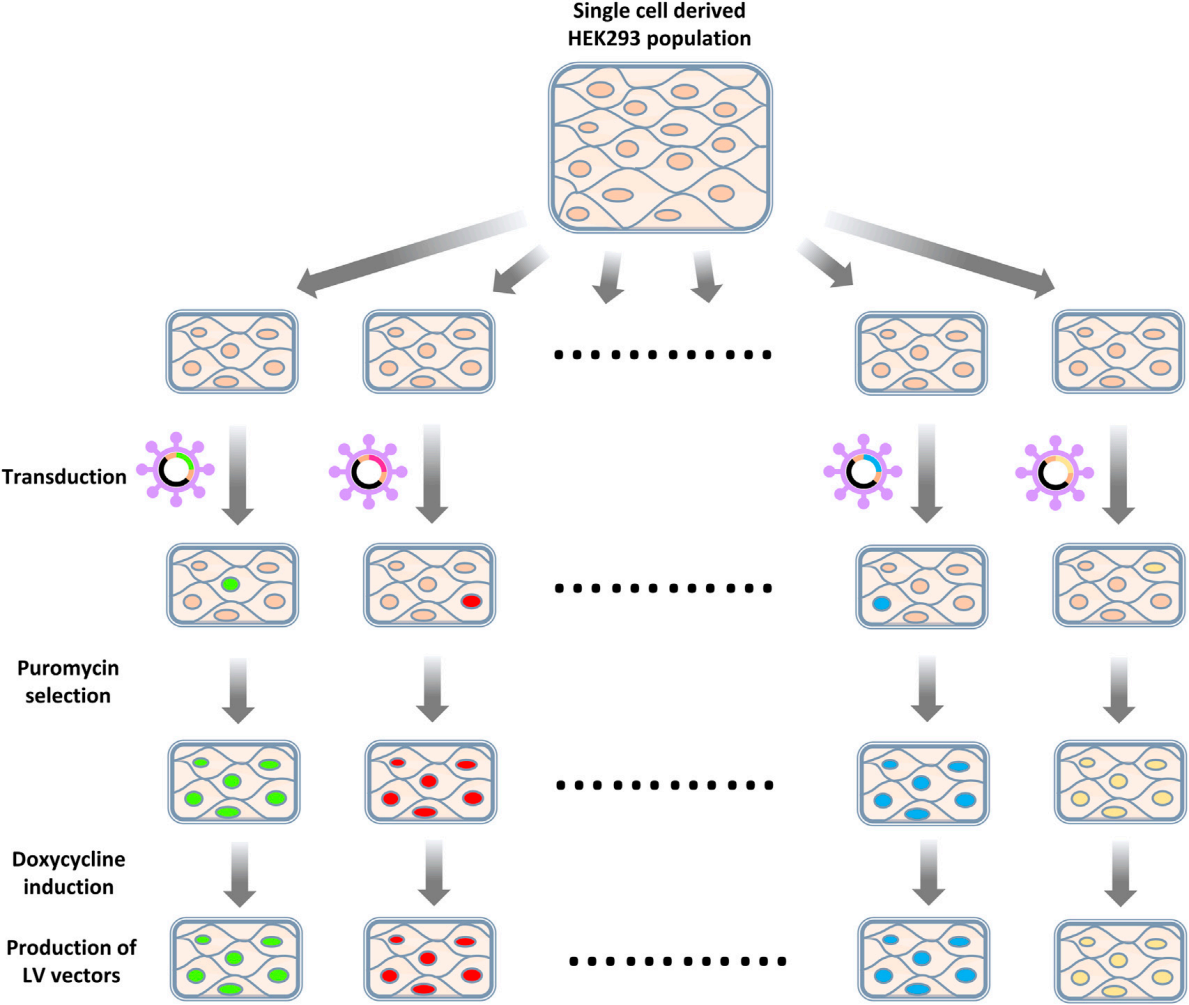
Workflow of knockout cell population generation. HEK293 cells were first transduced with LV vectors targeting selected exons or a control locus. Transduced cells were then selected by puromycin selection. Cas9 expression was induced by introducing doxycycline. After a recovery period, cells were co-transfected with an LV packaging plasmid and a VSV-G-encoding plasmid to assess the titers of LV vectors produced.

**FIGURE 5:**
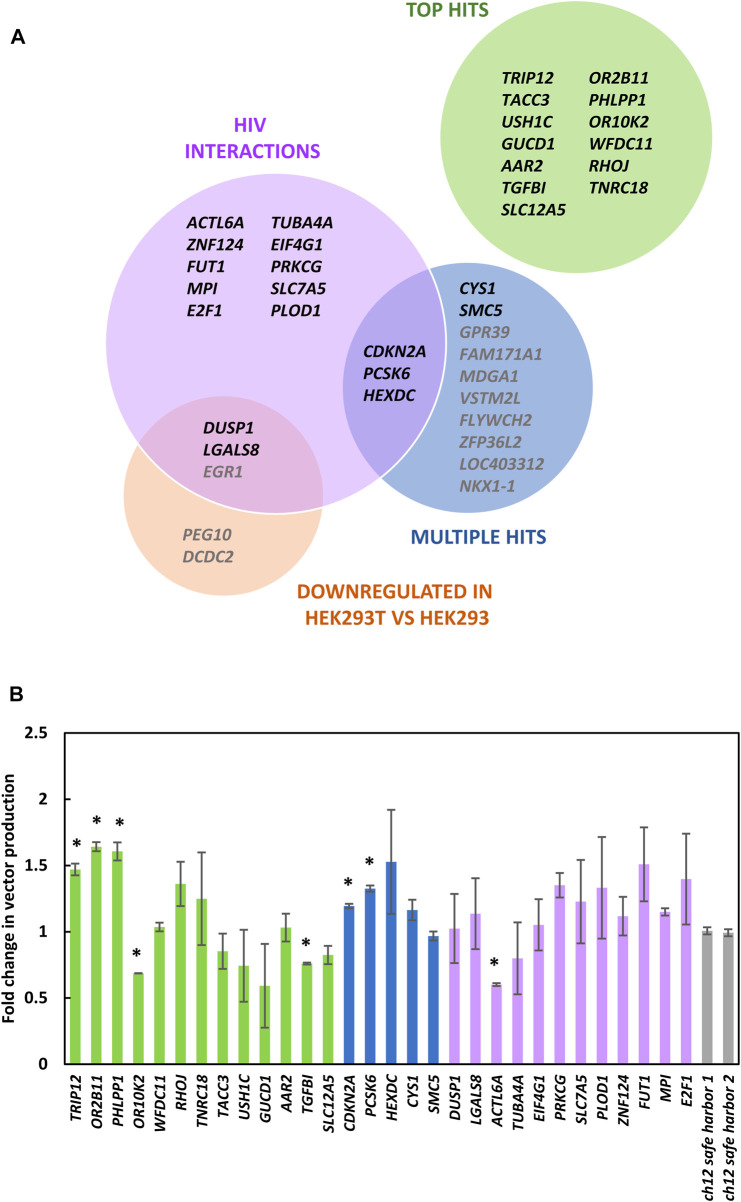
Characterization of bulk knockout populations. **(A)** 30 gRNAs enriched in the library screen that were selected for further characterization. The Top Hits category are the genes with individual gRNA that were most enriched. Multiple hits show genes that had multiple gRNAs within the top 500 of library hits. The HIV interactions category shows genes that had enriched gRNA within the top 1,000 of library members and may increase HIV titers when perturbed based on previously described HIV interactions (Ptak et al., 2008; Fu et al., 2009; Pinney et al., 2009). Genes downregulated in HEK293T vs. HEK293 lists genes downregulated by a factor of four or more within the top 1,000 library members (Lin et al., 2014). **(B)** LV vector titers of bulk knockout populations from panel **(A)** relative to cells transduced with gRNAs targeting a safe harbor locus (Chaikind et al., 2016). Genes from panel **(A)** listed in gray were not tested in panel **(B)**. Bars represent standard error of the mean of three biological replicates.

Enriched gRNAs were further characterized by generating knockout cell populations and observing the capacity of such cells to produce LV vectors (Figure 4). gRNAs were selected for this characterization based on their degree of enrichment, previously described interactions with HIV-1 that suggested a loss of gene function benefitted HIV productivity (Ptak et al., 2008; Fu et al., 2009; Pinney et al., 2009) and if multiple gRNAs targeting the same gene were enriched (Figure 5A). We elected to generate bulk knockout populations using the doxycycline inducible CRISPR/Cas9 LV vector, TLCV2-LoxP. This allowed for Cas9 nuclease expression and knockouts to be generated in response to doxycycline addition, and the testing of LV production in the absence of nuclease activity. This emulates the heterogeneity of knockouts that would be present during library screening, as in both cases a population of unedited cells and cells edited to varying degrees are established. Accordingly, we expected that increases in vector titers from gene knockouts using this method may be similar to those bioinformatically inferred from the initial library screening. A sequence on chromosome 12 was selected as a control target, such that control cells still experience Cas9 nuclease activity, but known genes were avoided (Chaikind et al., 2016).

Using this method knockout populations were generated for 30 of the gRNAs identified. As the most enriched gRNA targeted *TRIP12* (Gudjonsson et al., 2012), we characterized the editing efficiency achieved in the bulk *TRIP12* knockout population, revealing a knockout efficiency of 50% both by Next-Generation and Sanger Sequencing. The ability of these bulk populations to produce LV vectors was compared (Figure 5B). Among the most enriched gRNAs, the top three hits, targeting *TRIP12*, *OR2B11* and *PHLPP1* yielded significantly higher vector titers than cells transduced with control gRNAs. We observed that genes within the multiple gRNA hit category seemed to perform better than control cells most consistently, suggesting that this methodology would benefit from additional replicates with greater sequencing depth, to accommodate formal gene-level analysis.

### 3.4 Replicated knockout and activation screens in HEK293T cells

**FIGURE 6:**
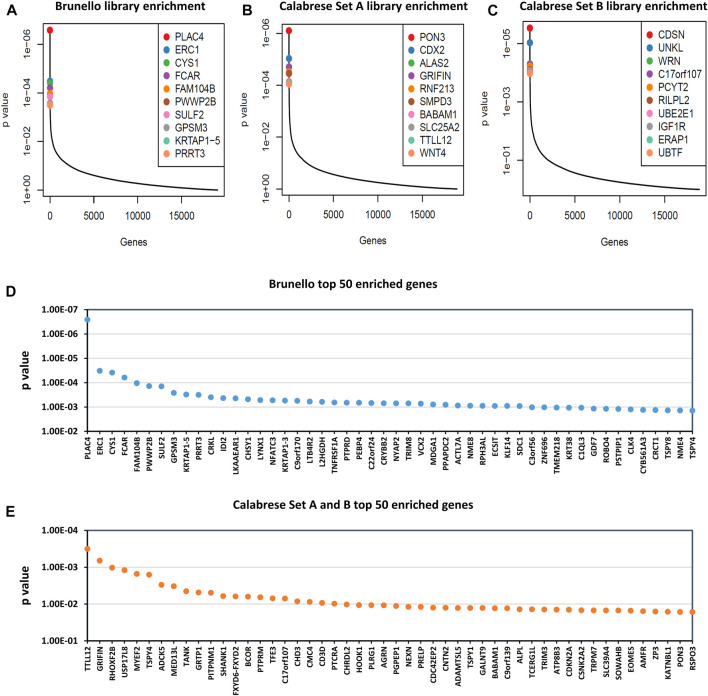
Gene enrichment of Brunello and Calabrese gRNA after 8 rounds of rescue transfection. Read counts were analyzed by MAGeCK (Li et al., 2014), where reads generated from cloned library plasmids were classed as controls and reads generated from viral supernatant were classed as treatment. Outputs of the most enriched genes are shown in plots: **(A)** Brunello library enrichment, **(B)** Calabrese Set A library enrichment and **(C)** Calabrese Set B library enrichment, where the *y*-axis indicates *p*-value and the *x*-axis represents genes ordered from most to least enriched. **(D)** Brunello library enrichment, showing top 50 most enriched genes on *x*-axis. **(E)** Combined Calabrese library enrichment, showing top 50 most enriched genes on *x*-axis and average *p*-value on the *y*-axis.

We went on to conduct larger scale, replicated screens using the Brunello and Calabrese libraries in HEK293T cells. We elected to use HEK293T cells as they are highly relevant to LV vector production and have much greater LV yields (Bae et al., 2020), allowing for the large number of transducing units required to be generated. The Brunello rescues typically yielded titers near 1 × 10^7^ TU/mL, while the Calabrese rescues yielded two to three x 10^6^ TU/mL. After sequencing, the enrichment of gRNAs targeting select genes was observed (Figure 6). The most enriched knockout target was *PLAC4*, while the most enriched activation target was *TTLL12*. In comparing the results of the pilot Brunello screen to the replicated screen in HEK293T cells, we observed that the top 10 gRNA from the “top hits” and “multiple hits” categories of the pilot averaged within the top 15% and 7.5% of enriched genes, respectively. Raw counts and gene-level analysis for replicated Brunello and Calabrese library screenings is available in Supplementary Table S3. Similarly, Gene Set Enrichment Analysis (GSEA) was carried out and is available in Supplementary Table S4.

## 4 Discussion

HEK293 derived cells were shown to yield higher vector titers compared to other cell lines such as African Green monkey-derived COS-7 cells (Reiser et al., 1996) and human TE571 cells (Chang et al., 1999). Notably, HEK293T cells, which contain SV40 large T-antigen sequences yield substantially higher LV vector titers than the parental cell line, HEK293 (Gama-Norton et al., 2011; Bae et al., 2020). The presence of T-antigen-encoding sequences has been shown to increase LV vector titers in HEK293 cells (Ferreira et al., 2020); The knockdown or removal of T-antigen-encoding sequences in HEK293Ts by short hairpin RNA (shRNA) or CRISPR/Cas9 respectively, were found to not appreciably decrease LV vector titers (Bae et al., 2020; Ferreira et al., 2020). This suggests that genetic perturbations created by SV40 large T-antigen may positively impact LV vector production. This phenomenon was repeated by the addition of a SV40 T-antigen sequence to HEK293 cells (Gama-Norton et al., 2011), confirming that SV40 T-antigen sequences underly the increase in LV vector titers. In addition, there are large-scale differences in chromosome copy number between HEK293 and HEK293T cells, such as a notable high degree of duplication of a region of chromosome 13 in HEK293T cells (Lin et al., 2014). This duplicated region contains the miRNA-17-92 cluster, which plays a pivotal role in many cellular processes (Mogilyansky and Rigoutsos, 2013). In addition, there are 155 upregulated and 334 downregulated genes in HEK293T vs. HEK293 (Lin et al., 2014), some of which may impact vector titers. Such genes represent potential rational targets to improve LV vector titers in HEK293 cells. Moreover, the large increase in LV vector titers resulting from genetic differences between HEK293 and HEK293T suggests that genetic perturbations that increase titer exist and may be identified by an unbiased approach.

While HEK293 and HEK293 cell derivatives are commonly used to produce LV vectors, there are many genetic improvements that increase LV vector production likely yet to be realized. Such cells contain cellular bottlenecks and viral restriction factors that may impact LV vector titers. In the case of HIV infection, many host restriction factors limit virus production and transduction (Zotova et al., 2019). These restriction factors are mitigated by viral accessory proteins, which are purposefully omitted in LV vectors due to safety concerns. As lentiviral vectors are HIV derived, it may be expected that restriction factors are present in human cell lines such as HEK293 (Uhlén et al., 2015). Expression data from the Human Protein Atlas (https://www.proteinatlas.org/about/licence) (Uhlén et al., 2015) suggests that most are expressed to varying degrees in HEK293 cells, with the notable exceptions of BST-2, Trim22, and Mx2. It was also recently reported that HEK293T cells lack an intact innate immune response, which may contribute to their amenability to LV vector production. However, while differences in titer when an innate immune response was or was not activated were observed, they did not reach statistical significance (Ferreira et al., 2020).

In our pilot gene knockout screen, we observed the regular enrichment of select gRNA throughout multiple rounds of rescue transfections. This affirmed that the changes observed in gRNA frequency had biological underpinnings and were not simply stochastic effects. When using such gRNA to generate gene knockouts in bulk populations, we were able to observe modest increases in LV vector titers for some gRNAs. To explore this approach more fully, we conducted additional screening in HEK293T cells using both knockout and activation libraries.

Gene-level analysis revealed that the most enriched knockout target was *PLAC4*. Interestingly, *PLAC4* is an RNA gene that has recently been implicated in interferon response and reduced covid-19 loads (Rouchka et al., 2021). In addition, gRNA targeting genes *FCAR, PRRT3* and *NFATC3*, which are downregulated by HIV-1 infection were enriched (Dürrbaum-Landmann et al., 1994). Lastly, genes that were identified in the pilot screen, *CYS1*, *CDKN2A, MDGA1,* and *VSTM2L*, were again enriched in the larger scale screening, suggesting they may have merit as potential targets to improve LV titers.

When combining the enrichment data for Calabrese Set A and Set B, we identified the most enriched activation target was *TTLL12*, which was ranked as the 9th and 30th most enriched gene within the two experiments. *TTLL12* has been shown to inhibit antiviral innate immunity (Ju et al., 2017). Along these lines, Gene Set Enrichment Analysis found significance enrichment of a gene set implicated in nucleic acid metabolism and innate immune sensing for both Calabrese Set A & B (*p* ≤ 0.1) and for the Brunello data (*p* ≤ 0.05). Moreover, *TTLL12* is also upregulated by HIV-1 Tat (Jarboui et al., 2012). In addition to *TTLL12*, gRNAs targeting the three genes *MYEF2*, *GRTP1*, and *TFE3*, were highly enriched. The knockdown of these genes has been demonstrated to diminish HIV-1 replication (Brass et al., 2008; König et al., 2008).

One potential source of false positives in this workflow is caused by using a combined (one-vector) system, expressing both Cas9 and a gRNA (Sanjana et al., 2014). This may result in vectors lacking expression of Cas9 due to inactivation or deletion of Cas9 sequence. Both events would likely be interpreted as an increase in vector titers, as cells without Cas9 would be expected to proliferate faster and since smaller vector genomes may result in higher vector titers. To mitigate this potential shortcoming, when conducting our large-scale screening, we proceeded with a two-vector system where cell lines are made to stably express Cas9 prior to initiating screening. Similarly, another important consideration in this workflow when conducting gene knockouts, is the limited exposure of cells to Cas nuclease reagents. This limited exposure may be insufficient to yield a phenotypic effect, depending on factors such as the efficiency of each gRNA, the length of the indels introduced, the copy number of the targeted gene and mRNA and protein turnover rates of a given gene. Using a two-vector system, where cells are already producing Cas9 nuclease prior to transduction may increase the extent of editing. Similarly, this approach may benefit from screening in cell lines with reduced ploidy levels. However, this is less of a concern for activation screening. Given the potential for truncated vectors to emerge that still contain gRNA sequences that could be amplified, biological replicates were used to control for such rare events where, for example, a vector harboring a given gRNA may gain a mutation that increases productivity. By including replicates, it would become unlikely that a given gRNA would independently acquire such mutations multiple times. While beyond the scope of this paper, it would be worthwhile to generate full length reads of library vectors and observe the abundance of such mutations over time. Such analysis could potentially be used after screening to remove false positives.

We expect that some hits identified using approaches similar to those described here may be specific to a given experimental setup. Library screening using different vector pseudotypes, cell lines or culture conditions could potentially yield different results. It is also notable that this approach involved the use of a second-generation packaging plasmid encoding Tat during transfection, while applications of screening would most likely be utilized in a third-generation context. Additionally, library screening could be conducted where vectors harboring gRNA also carry a transgene of interest to probe for transgene specific responses that reduce titers. As there are currently gRNA libraries for gene knockout, inhibition and activation, as well as libraries targeting long non-coding RNA (Zhu et al., 2016), there is much potential yet to be realized for improved cell lines for LV vector production.

In the future, we imagine that libraries that delete large regions of genomic DNA using CRISPR Cascade will become available (Dolan et al., 2019). Events from several CRISPR library variants could potentially be stacked to identify improved cell lines for LV vector production. Here we have listed several knockout and overexpression candidate genes that may be used to rationally develop improved LV production cell lines in the future. Furthermore, our results are a first step that demonstrates a feasible, unbiased workflow to identify genetic modifications that improve LV vector titers.

## Data Availability

The plasmids presented in the study are deposited in addgene.org, Addgene Plasmid #173428, #182635, #182646 and #182647.
