# Supplementary material for: CRISPR library screening to develop HEK293-derived cell lines with improved lentiviral vector titers
Source: Front Genome Ed. 2023 Jul 13;5:1218328. doi: 10.3389/fgeed.2023.1218328 (PMC10373892; doi:10.3389/fgeed.2023.1218328)
Supplement: Supplementary file 3 [file Table1.docx]

Supplementary Material

CRISPR Library Screening to Develop Cell Lines with Improved Lentiviral Vector Titers

Brian Iaffaldano*, Michael P. Marino, Jakob Reiser

*** Correspondence:** Brian Iaffaldano: brian.iaffaldano@gmail.com

# Supplementary Data

**Supplementary Figure S1.** Sequence of gBlock WPRE wt3'LTR

**Supplementary Figure S2.** Homozygous knockout of *TRIP12* gene through RNP transfection. (A) A diagram showing the competitive PCR assay for mutation detection using three primers F (5’ TGAGGTTGGGAGTTGGAGAC 3’), R1 (5’ AAAAGACCACGGGCTCCTG 3’) and R2 (5’ ACCGATCCTGAACTGGAGTG). (B) Amplicons are predominantly 368 bp for wild type cells as small products are preferred in the competitive PCR assay. In cells with *TRIP12* sequences homozygously knocked out, primer R1 binding site is disrupted and only the 643 bp PCR product is observed. WT, wild type. L, NEB 1kb Plus Ladder. (C) Expected genotype from Sanger sequencing results of a *TRIP12* homozygous knockout cell clone. The protospacer sequence is highlighted in red while the PAM sequence is highlighted in blue.

**Supplementary Table S1.** Oligonucleotides used in this study

**Supplementary Table S2.** Provides gRNA count data for pilot Brunello screening in HEK293 cells. Normalized counts for the Brunello library categories described in Figures 2 and 3 are provided, where each column sums to 1. Additional data for Rescue rounds 5 and 6 to compare methodologies is provided, where amplicons were produced using a reverse transcription step followed by a high-fidelity polymerase (Q5), as opposed to a one-pot method (OneTaq® One-Step RT-PCR Kit), which was used throughout.

**Supplementary Table S3.** Provides raw counts for larger scale screening of Brunello and Calabrese (Set A and Set B) libraries in HEK293T cells. Gene-level analysis of positive selection is also provided for screening of Brunello and Calabrese (Set A and Set B) libraries. The combined gene-level analysis for Calabrese Set A and Set B is also provided, where the average p-value for each gene was calculated with Set A and Set B weighted equally.

**Supplementary Table S4.** Provides Gene Set Enrichment Analysis of Brunello and Calabrese (Set A and Set B) libraries in HEK293T cells using the C2 gene Sets (https://www.gsea-msigdb.org/gsea/msigdb/human/genesets.jsp?collection=C2).

**5’-GCGTTAAGTCGACAATCAACCTCTGGATTACAAAATTTGTGAAAGATTGACTGGTATTCTTAACTATGTTGCTCCTTTTACGCTATGTGGATACGCTGCTTTAATGCCTTTGTATCATGCTATTGCTTCCCGTATGGCTTTCATTTTCTCCTCCTTGTATAAATCCTGGTTGCTGTCTCTTTATGAGGAGTTGTGGCCCGTTGTCAGGCAACGTGGCGTGGTGTGCACTGTGTTTGCTGACGCAACCCCCACTGGTTGGGGCATTGCCACCACCTGTCAGCTCCTTTCCGGGACTTTCGCTTTCCCCCTCCCTATTGCCACGGCGGAACTCATCGCCGCCTGCCTTGCCCGCTGCTGGACAGGGGCTCGGCTGTTGGGCACTGACAATTCCGTGGTGTTGTCGGGGAAATCATCGTCCTTTCCTTGGCTGCTCGCCTGTGTTGCCACCTGGATTCTGCGCGGGACGTCCTTCTGCTACGTCCCTTCGGCCCTCAATCCAGCGGACCTTCCTTCCCGCGGCCTGCTGCCGGCTCTGCGGCCTCTTCCGCGTCTTCGCCTTCGCCCTCAGACGAGTCGGATCTCCCTTTGGGCCGCCTCCCCGCGTCGACTTTAAGACCAATGACTTACAAGGCAGCTGTAGATCTTAGCCACTTTTTAAAAGAAAAGGGGGGACTGGAAGGGCTAATTCACTCCCAACGAAGACAAGATATCCTTGATCTGTGGATCTACCACACACAAGGCTACTTCCCTGATTGGCAGAACTACACACCAGGGCCAGGGATCAGATATCCACTGACCTTTGGATGGTGCTACAAGCTAGTACCAGTTGAGCAAGAGAAGGTAGAAGAAGCCAATGAAGGAGAGAACACCCGCTTGTTACACCCTGTGAGCCTGCATGGGATGGATGACCCGGAGAGAGAAGTATTAGAGTGGAGGTTTGACAGCCGCCTAGCATTTCATCACATGGCCCGAGAGCTGCATCCGGAGTACTTCAAGAACTGCTGACATCGAGCTTGCTACAAGGGACTTTCCGCTGGGGACTTTCCAGGGAGGCGTGGCCTGGGCGGGACTGGGGAGTGGCGAGCCCTCAGATGCTGCATATAAGCAGCTGCTTTTTGCTTGTACTGGGTCTCTCTGGTTAGACCAGATCTGAGCCTGGGAGCTCTCTGGCTAACTAGGGAACCCACTGCTTAAGCCTCAATAAAGCTTGCCTTGAGTGCTTCAAGTAGTGTGTGCCCGTCTGTTGTGTGACTCTGGTAACTAGAGATCCCTCAGACCCTTTTAGTCAGTGTGGAAAATCTCTAGCAGGGCCCGTTTAAACCCGCTGATCAGCCTCGACTGT**

**-3’**

**Supplementary Figure S1.** Sequence of gBlock WPRE wt3'LTR


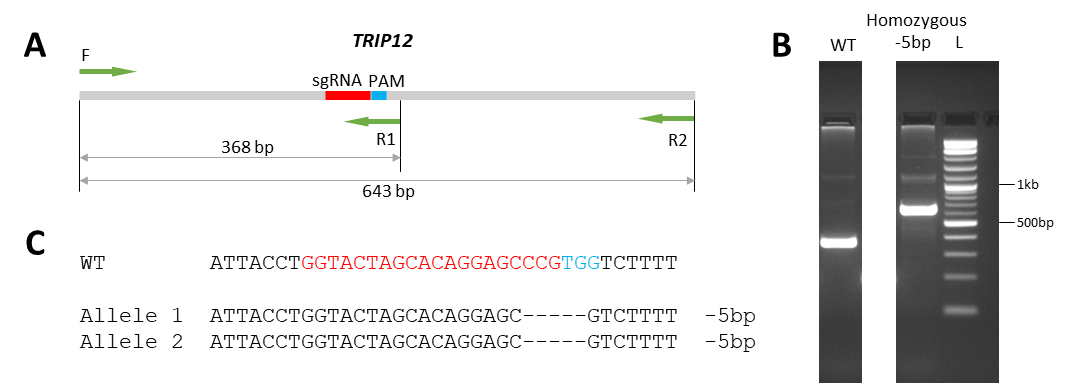


**Supplementary Figure S2.** Homozygous knockout of *TRIP12* gene through RNP transfection. (A) A diagram showing the competitive PCR assay for mutation detection using three primers F (5’ TGAGGTTGGGAGTTGGAGAC 3’), R1 (5’ AAAAGACCACGGGCTCCTG 3’) and R2 (5’ ACCGATCCTGAACTGGAGTG 3’). (B) Amplicons are predominantly 368 bp for wild type cells Sas small products are preferred in the competitive PCR assay. In cells with *TRIP12* sequences homozygously knocked out, primer R1 binding site is disrupted and only the 643 bp PCR product is observed. WT, wild type. L, NEB 1kb Plus Ladder. (C) Expected genotype from Sanger sequencing results of a *TRIP12* homozygous knockout cell clone. The protospacer sequence is highlighted in red while the PAM sequence is highlighted in blue.

**Supplementary Table S1.** Oligonucleotides used in this study

| Primer Name | Sequence 5’ - 3’ |
| --- | --- |
| AAR2 F | CACCGGGGGTAGATTCTGCCCCACG |
| AAR2 R | AAACCGTGGGGCAGAATCTACCCCC |
| ACTL6A F | CACCGAGAAGTTGCCTCAGGTTACG |
| ACTL6A R | AAACCGTAACCTGAGGCAACTTCTC |
| CDKN2A F | CACCGCACCGAATAGTTACGGTCGG |
| CDKN2A R | AAACCCGACCGTAACTATTCGGTGC |
| CYS1 F | CACCGCTGGCCGAGTCGGCGGCCTG |
| CYS1 R | AAACCAGGCCGCCGACTCGGCCAGC |
| DUSP1 F | CACCGGCAAACAGTCGACCCCCATG |
| DUSP1 R | AAACCATGGGGGTCGACTGTTTGCC |
| E2F1 F | CACCGCTGGATGCCCTCAAGGACGT |
| E2F1 R | AAACACGTCCTTGAGGGCATCCAGC |
| EIF4G1 F | CACCGGGATCTCGAATTCGGATCTG |
| EIF4G1 R | AAACCAGATCCGAATTCGAGATCCC |
| FUT1 F | CACCGTGGACGCCGACAAAGGTGCG |
| FUT1 R | AAACCGCACCTTTGTCGGCGTCCAC |
| GUCD1 F | CACCGAGCGGTGCCTCACGCCAAAG |
| GUCD1 R | AAACCTTTGGCGTGAGGCACCGCTC |
| HEXDC F | CACCGGTGCCTTCAAGGGTGCCACG |
| HEXDC R | AAACCGTGGCACCCTTGAAGGCACC |
| LGALS8 F | CACCGTGAAAGGCCACATCGGCTCG |
| LGALS8 R | AAACCGAGCCGATGTGGCCTTTCAC |
| MPI F | CACCGAGGATCTTGGCATCCCCTCG |
| MPI R | AAACCGAGGGGATGCCAAGATCCTC |
| OR10K2 F | CACCGACTCAGTGCTAATGGGACAT |
| OR10K2 R | AAACATGTCCCATTAGCACTGAGTC |
| OR2B11 F | CACCGACTCCGTGCATCCCAGCCAG |
| OR2B11 R | AAACCTGGCTGGGATGCACGGAGTC |
| PCSK6 F | CACCGCAAGAACAGTCGCTGCCGGT |
| PCSK6 R | AAACACCGGCAGCGACTGTTCTTGC |
| PHLPP1 F | CACCGACTCGGAGGTACCGCCCGCG |
| PHLPP1 R | AAACCGCGGGCGGTACCTCCGAGTC |
| PLOD1 F | CACCGATGACGTGCTGTTTGCATCG |
| PLOD1 R | AAACCGATGCAAACAGCACGTCATC |
| PRKCG F | CACCGACTCGAAGGTCACAAATTCG |
| PRKCG R | AAACCGAATTTGTGACCTTCGAGTC |
| RHOJ F | CACCGGCTCGGACTGTATGACACCG |
| RHOJ R | AAACCGGTGTCATACAGTCCGAGCC |
| SLC12A5 F | CACCGACGGTGACCACACGGCTATG |
| SLC12A5 R | AAACCATAGCCGTGTGGTCACCGTC |
| SLC7A5 F | CACCGCGACTACGCCTACATGCTGG |
| SLC7A5 R | AAACCCAGCATGTAGGCGTAGTCGC |
| SMC5 F | CACCGCCAACGATCATATTCAAGTG |
| SMC5 R | AAACCACTTGAATATGATCGTTGGC |
| TACC3 F | CACCGGACTTGGTGTCACCTCCGAA |
| TACC3 R | AAACTTCGGAGGTGACACCAAGTCC |
| TGFBI F | CACCGGCTCGGCTTACCCGTGCTGG |
| TGFBI R | AAACCCAGCACGGGTAAGCCGAGCC |
| TNRC18 F | CACCGGCTCGGGCAACGCATCCATG |
| TNRC18 R | AAACCATGGATGCGTTGCCCGAGCC |
| TRIP12 F | CACCGGGTACTAGCACAGGAGCCCG |
| TRIP12 R | AAACCGGGCTCCTGTGCTAGTACCC |
| TUBA4A F | CACCGGAGCCGCTCCATCAGGAGTG |
| TUBA4A R | AAACCACTCCTGATGGAGCGGCTCC |
| USH1C F | CACCGCAGCAGAGGAAATCTGCTCG |
| USH1C R | AAACCGAGCAGATTTCCTCTGCTGC |
| WFDC11 F | CACCGACGGTGCTACTGTCTGTGCT |
| WFDC11 R | AAACAGCACAGACAGTAGCACCGTC |
| ZNF124 F | CACCGAAACAACCCATATGGGTGTG |
| ZNF124 R | AAACCACACCCATATGGGTTGTTTC |
| ch12 safe harbor 1-F | CACCGACTTTTTGGGGGAGAGGGAG |
| ch12 safe harbor 1-R | AAACCTCCCTCTCCCCCAAAAAGTC |
| ch12 safe harbor 2-F | CACCGAAATAGCACAATGAATGGAA |
| ch12 safe harbor 2-R | AAACTTCCATTCATTGTGCTATTTC |
